# Mitochondrial Lon sequesters and stabilizes p53 in the matrix to restrain apoptosis under oxidative stress via its chaperone activity

**DOI:** 10.1038/s41419-018-0730-7

**Published:** 2018-06-13

**Authors:** Ya-Ju Sung, Ting-Yu Kao, Cheng-Liang Kuo, Chi-Chen Fan, An Ning Cheng, Wei-Cheng Fang, Han-Yu Chou, Yu-Kang Lo, Chung-Hsing Chen, Shih Sheng Jiang, I-Shou Chang, Chun-Hua Hsu, Jin-Ching Lee, Alan Yueh-Luen Lee

**Affiliations:** 1 National Institute of Cancer Research, National Health Research Institutes, Zhunan, Miaoli 35053 Taiwan; 2 Department of Medical Laboratory Science and Biotechnology, Yuanpei University of Medical Technology, Hsinchu, 300 Taiwan; 3 Superintendent Office, Mackay Memorial Hospital, Taipei, Taiwan; 4 Genome and Systems Biology Degree Program, National Taiwan University and Academia Sinica, Taipei, 10617 Taiwan; 5 Department of Agricultural Chemistry, National Taiwan University, Taipei, 10617 Taiwan; 6 Department of Biotechnology, College of Life Science, Kaohsiung Medical University, Kaohsiung, Taiwan; 7 Graduate Institute of Medicine, College of Medicine, Kaohsiung Medical University, Kaohsiung, Taiwan; 8 Department of Medical Research, Kaohsiung Medical University Hospital, Kaohsiung, Taiwan

## Abstract

Mitochondrial Lon is a multi-function matrix protease with chaperone activity. However, little literature has been undertaken into detailed investigations on how Lon regulates apoptosis through its chaperone activity. Accumulating evidences indicate that various stresses induce transportation of p53 to mitochondria and activate apoptosis in a transcription-independent manner. Here we found that increased Lon interacts with p53 in mitochondrial matrix and restrains the apoptosis induced by p53 under oxidative stress by rescuing the loss of mitochondrial membrane potential (Δψm) and the release of cytochrome C and SMAC/Diablo. Increased chaperone Lon hampers the transcription-dependent apoptotic function of p53 by reducing the mRNA expression of p53 target genes. The ATPase mutant (K529R) of chaperone Lon decreases the interaction with p53 and fails to inhibit apoptosis. Furthermore, the chaperone activity of Lon is important for mitochondrial p53 accumulation in an mtHsp70-dependent manner, which is also important to prevent the cytosolic distribution of p53 from proteasome-dependent degradation. These results indicate that the chaperone activity of Lon is important to bind with mitochondrial p53 by which increased Lon suppresses the apoptotic function of p53 under oxidative stress. Furthermore, mitochondrial Lon-mtHsp70 increases the stability/level of p53 through trafficking and retaining p53 in mitochondrial matrix and preventing the pool of cytosolic p53 from proteasome-dependent degradation in vitro and in clinic.

## Introduction

The tumor-suppressor gene p53 is a key regulator of cell cycle arrest, senescence, and cell death including apoptosis and necrosis^1–3^. Thus p53 acts as one of the most important barriers against malignant development of cancer cells by linking many stress response pathways such as DNA damage, hypoxia, and oxidative stress^4^. A well-characterized function of p53 in the apoptosis regulation is its role as a transcriptional regulator. In addition to the functions as a transcription factor, p53 acts directly upon the outer membrane of mitochondria via a transcription-independent pathway. Upon onset of apoptosis following DNA damage stress, a part of p53 translocates to mitochondria, where it interacts with Bcl-2 or Bak, resulting in cytochrome C release and caspase-3 activation^5^. In addition, p53 accumulates in the mitochondrial matrix and triggers mitochondrial permeability transition pore (MPTP) opening and necrosis by interaction with the MPTP regulator cyclophilin D under oxidative stress^2^. However, mechanisms of p53-mediated transcription-independent apoptotic pathways in mitochondrial matrix are still lacking.

Mitochondria control cell death and survival by regulating intrinsic apoptosis, autophagy, necrosis, and ferroptosis^2,6,7^. Mitochondrial Lon protease is located in matrix and plays a crucial role in the maintenance of mitochondrial function, biogenesis, and homeostasis^8,9^. In addition to its ATP-dependent proteolytic activity, mitochondrial Lon has been found to show chaperone activity^10–13^. Mitochondrial Lon is a stress protein and induced by a number of stresses, such as hypoxia, oxidative, and unfolded protein stress^10,12,14,15^. Molecular chaperones including mitochondrial chaperones have been associated with enhanced cell survival under stress by inhibition of apoptotic cell death and increased stability of survival effectors that promote tumor growth^16–18^. Indeed, Lon downregulation causes loss of mitochondrial function, early embryonic lethality, reduced cell proliferation, and apoptosis^12,19–21^. Lon upregulation is required for cancer cell survival and tumorigenesis by regulating stress responses induced by oxidative condition^12,20,22^. However, the molecular mechanism of how Lon regulates apoptosis remains largely unclear. We recently identified heat-shock protein 60 (Hsp60) and mitochondrial Hsp70 (mtHsp70) as chaperone Lon clients by utilizing proteomic approach^17^. Interestingly, the ability of increased Lon-inhibited apoptosis is dependent on Hsp60 that binds p53 to inhibit apoptosis^16,17^. These findings allowed us to pursue the detailed mechanism of how chaperone Lon directly regulates apoptosis by interacting with p53.

To our knowledge, the present study for the first time demonstrates that p53 is bound by Lon in the mitochondrial matrix to control apoptosis. In this study, we demonstrated that Lon interacts with p53 in mitochondrial matrix and restrains the apoptosis induced by p53 under oxidative stress by reducing the mRNA expression of p53 target genes and rescuing the loss of mitochondrial membrane potential (Δψm) and the release of cytochrome C. The ATPase mutant (K529R) of mitochondrial Lon decreased the interaction with p53, reduced mitochondrial localization of p53, and failed to inhibit apoptosis, suggesting that the chaperone activity of Lon is important for the control of p53 protein level and apoptotic function by sequestering p53 in mitochondrial matrix. In addition, the level of cytoplasmic p53 significantly correlates that of mitochondrial Lon in oral cancer patients. Thus our findings suggest that targeting the chaperone activity of mitochondrial Lon will increase the efficacy of p53-induced apoptosis in cancer therapy.

## Results

### Overexpression of mitochondrial Lon increases the accumulation of mitochondrial p53 and restrains p53-dependent apoptosis under oxidative stress

**Fig. 1:**
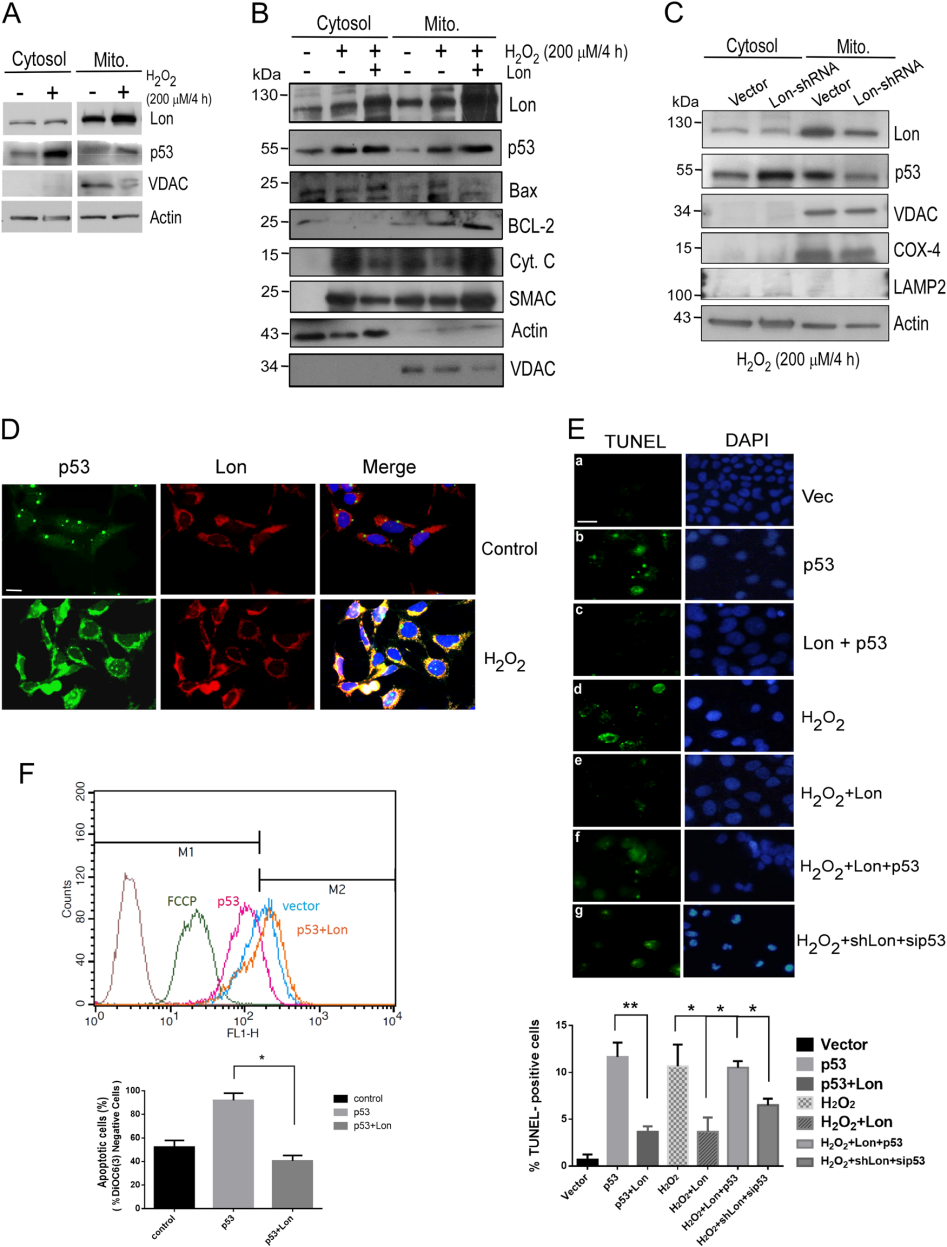
Increased Lon increases the accumulation of mitochondrial p53 and inhibits p53-dependent apoptosis under oxidative stress in cancer cells. **a** Immunoblotting analysis of the increase of p53 and Lon in the mitochondrial fraction under oxidative stress. HSC3 cells were treated with 200 μM H_2_O_2_ for 4 h. Immunoblotting were performed using the indicated antibodies. The purity of each cell fractions was monitored by immunoblotting for cytoplasmic (Actin) and mitochondrial (VDAC) markers. **b** Increased mitochondrial Lon restrains p53-dependent apoptosis in cancer cells under oxidative stress. HSC3 cells transfected with or without pcDNA3-Myc-Lon were treated with 200 μM H_2_O_2_ for 4 h, then recovered for 4 h. Whole-cell lysates were used to purify the mitochondrial and cytosolic fractions. Apoptosis-associated proteins were detected by western blot analysis using the indicated antibodies. The purity of mitochondrial fraction was monitored by immunoblotting for mitochondrial (VDAC) markers, and anti-Actin and anti-VDAC antibodies were used as loading control. **c** Mitochondrial Lon is required for mitochondrial accumulation of p53 under oxidative stress. HSC3 cells were transfected with the plasmid encoding Lon-shRNA. The transfected cells were treated with 200 μM H_2_O_2_ for 4 h. Immunoblotting were performed using the indicated antibodies. The purity of each cell fractions was monitored by immunoblotting for cytoplasmic (Actin), lysosome (LAMP2), and mitochondrial (VDAC and COX-4) markers. **d** The accumulation of mitochondrial Lon and p53 in mitochondria under oxidative stress was verified by immunofluorescence. 293T cells were untreated or treated with 50 μM H_2_O_2_ for 24 h. The cells were fixed and immunostained by anti-p53 (green) and anti-Lon (red) antibodies. DNA was stained with DAPI (blue). Scale bar, 10 μm. **e** Increased mitochondrial Lon restrains p53-dependent apoptosis under oxidative stress shown by TUNEL assay. The apoptosis in HSC3 cells was induced by p53 overexpression or by treatment with 200 μM H_2_O_2_ for 4 h. The treated HSC3 cells were transfected with pcDNA3-Myc-Lon, Lon-shRNA, p53-siRNA or not. Terminal deoxynucleotidyl transferase-mediated dUTP nick-end labeling (TUNEL) assay was applied to examine the effect of Lon overexpression on p53-induced apoptosis. TUNEL-positive cells (green fluorescence) were counted in the cells expressing vector (**a**), p53 overexpression (**b**), or Lon plus p53 (**c**). Treatment with H_2_O_2_ acted as a positive control (**d, e**). Treatment with H_2_O_2_ plus Lon and p53 (**f**) and with H_2_O_2_ plus Lon-shRNA and p53-siRNA (**g**). DAPI was used for nuclear staining. Scale bar, 20 μm. The error bars shown in the bottom panel represent the standard deviation from three different experiments. *p < 0.05 and **p < 0.01. **f** Increased mitochondrial Lon restrains p53-dependent apoptosis shown by mitochondrial membrane potential assay. The apoptosis in HSC3 cells was induced by p53 overexpression. The HSC3 cells were transfected with pcDNA3-Myc-Lon plasmid or not. Mitochondrial membrane potential assay was applied to examine the effect of Lon overexpression on p53-induced apoptosis. The transfected cells were stained with 50 nM DiOC6(3) for 30 min and analyzed with flow cytometer. FCCP treatment acts as a control of mitochondrial membrane potential which shows that the stain is fundamentally dependent on mitochondrial membrane potential. The error bars shown in the bottom panel represent the standard deviation from three different experiments. *p < 0.05

We previously showed that mitochondrial Lon physically interacts with Hsp60–mtHsp70 complex and regulates apoptosis through Hsp60^17^. Since Hsp60 binds p53 to restrain its apoptosis function in cytosol and mitochondria^16^, we asked whether Lon regulates p53-induced apoptosis under stress. We first found that the level of Lon and p53 are increased in cytosol and mitochondria after H_2_O_2_ and rotenone treatment (Fig. 1a, b, and Supplemental Figure S1). The level of cytosolic and mitochondrial p53 was further increased when Lon was overexpressed in cells (Fig. 1b) and only mitochondrial p53 was decreased when Lon was downregulated under oxidative stress (Fig. 1c), suggesting that the mitochondrial localization of p53 is regulated by Lon under oxidative stress. Since p53 protein can translocate to mitochondrial matrix in response to oxidative stress^2^, we tried to entangle the mitochondrial localization of p53 with Lon under oxidative stress. The immunofluorescence analysis showed that there was no significant colocalization of p53 (green) and Lon (red) in the control cells. Following exposure to H_2_O_2_, both p53 and Lon accumulated in the mitochondria, and they appeared colocalized with each other as yellow spots (Fig. 1d), which raised a possibility of the interaction between Lon and p53 in mitochondria under oxidative stress. Next we found that the signals of pro-apoptotic proteins, Bax, p53, cytochrome C release, and SMAC/Diablo release from mitochondria, are increased after H_2_O_2_ treatment. However, the activation of apoptosis was almost inhibited when Lon was overexpressed including the reduction of cytochrome C release (Fig. 1b). The results suggest that increased Lon restrains p53-induced apoptosis during oxidative stress. To confirm the role of Lon in p53-mediated apoptosis, terminal deoxynucleotidyl transferase-mediated dUTP nick-end labeling (TUNEL) assay was performed. The TUNEL-positive cells were detected more when the cells were overexpressed by p53 and treated with H_2_O_2_, compared to the vector control cells (a, b, and d in Fig. 1e). However, the TUNEL-positive signals in p53-overexpressed cells and the H_2_O_2_-treated cells were decreased when Lon was also overexpressed (c and e in Fig. 1e). We further found that the positive signals repressed by Lon overexpression were reversed when p53 was overexpressed (f in Fig. 1e). Consistently, the TUNEL-positive signals induced by decreased Lon were rescued when p53 was knocked down under H_2_O_2_ treatment (g in Fig. 1e). The mitochondrial membrane potential (MMP) analysis showed that MMP was decreased in p53-overexpressed cells, compared to the vector control cells, and the MMP in p53-overexpressed cells was rescued when Lon was overexpressed (Fig. 1f). These data showed that increased Lon protein restrains p53-dependent apoptosis in cancer cells under oxidative stress, in which Lon is required for mitochondrial p53 accumulation.

### Chaperone Lon interacts with p53 in mitochondrial fraction under oxidative stress

**Fig. 2:**
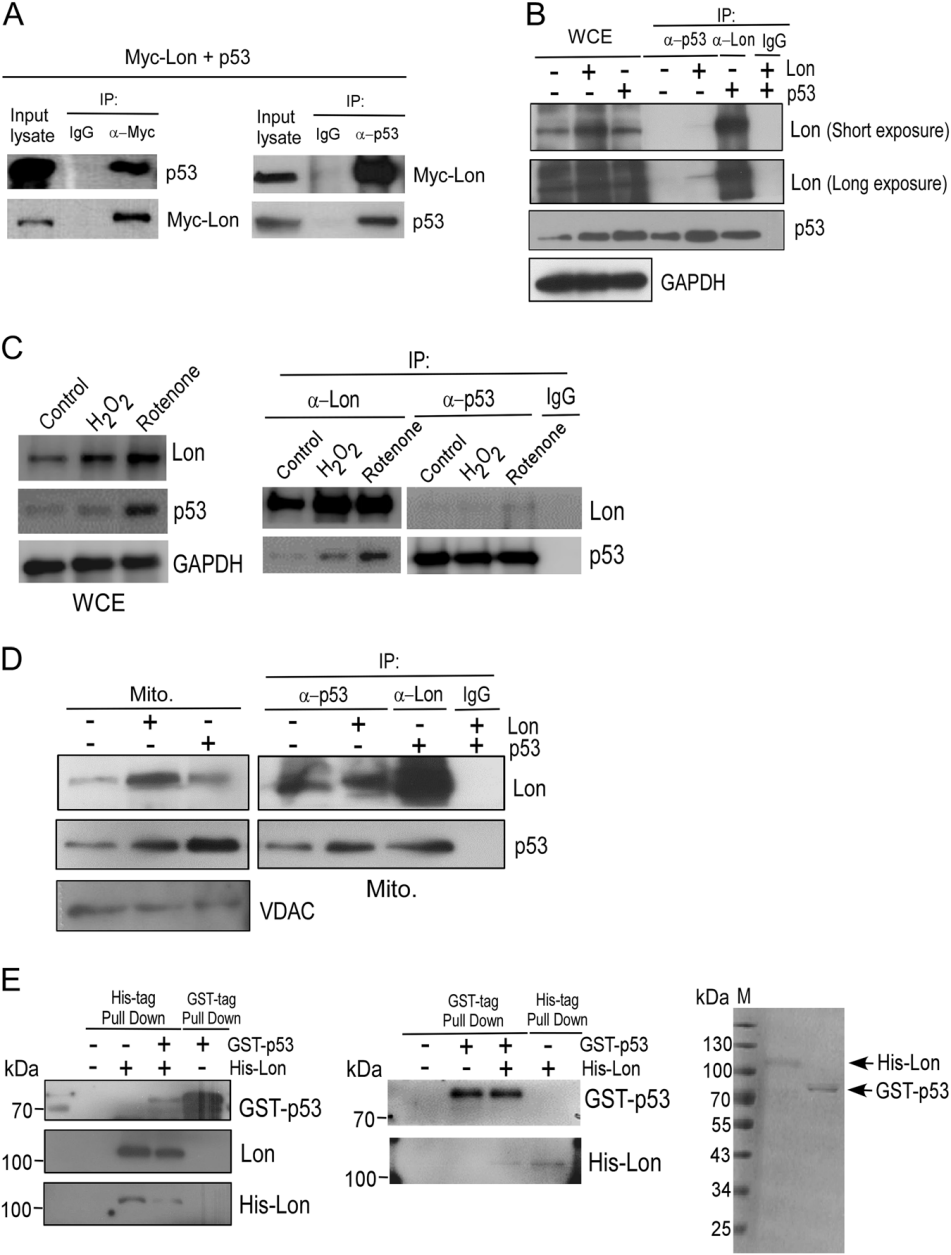
Chaperone Lon interacts with p53 in mitochondrial fraction under oxidative stress. **a**, **b** Lon interacts with p53 shown by co-immunoprecipitation. 293T (**a**) cells were transiently transfected with the plasmids encoding Myc-Lon and p53 followed by co-immunoprecipitation with anti-Myc and anti-p53, respectively. Whole-cell lysates from HSC3 (**b**) cells transfected with the plasmids encoding Myc-Lon or p53 were immunoprecipitated with anti-p53 or anti-Lon antibodies. The immunoprecipitation complex was analyzed by western blotting using the indicated antibodies. IP immunoprecipitation. **c** Lon interacts with endogenous p53 under oxidative stress shown by co-immunoprecipitation. HSC3 cells were treated with 2 mM H_2_O_2_ for 6 h or 2 μM rotenone for 6 h. Whole-cell lysates were analyzed by western blotting using the indicated antibodies (left panel). Whole-cell lysates from HSC3 cells treated with H_2_O_2_ or rotenone were immunoprecipitated with anti-p53 or anti-Lon antibodies. The immunoprecipitation complex was analyzed by western blotting using the indicated antibodies. **d** Lon interacts with p53 in mitochondrial fraction shown by co-immunoprecipitation. HSC3 cells were transiently transfected with the plasmids encoding Myc-Lon or p53. Whole-cell lysates from HSC3 cells were used to purify the mitochondrial and cytosolic fractions followed by co-immunoprecipitation with anti-p53 and anti-Myc, respectively. The mitochondrial fraction and the immunoprecipitation complex from transfected HSC3 cells were subjected to immunoblotting using the indicated antibodies. The purity of mitochondrial fraction was monitored by immunoblotting for mitochondrial (VDAC) markers. **e** Mitochondrial Lon interacts with p53 shown by the pull-down assay. Direct interaction between mitochondrial Lon and p53 was verified by His-tag and GST-tag pull-down assay. The His-Lon fusion protein and GST-p53 were added, and the His-tag proteins were pulled down by anti-His antibody and the GST-tag proteins were pulled down by anti-GST antibody. The pulled down complex was subjected to immunoblotting using the indicated antibodies. The His-tag/GST-tag proteins were pulled down by anti-His/anti-GST antibody as a positive control. The His- and GST-fusion proteins added were shown on SDS/PAGE stained with Coomassie brilliant blue (right panel)

Since Lon physically interacts with Hsp60–mtHsp70 to restrain its apoptosis function in mitochondria, we asked whether p53 is a direct client protein of chaperone Lon. The association between Lon and p53 was first examined by co-immunoprecipitation (Co-IP) experiment (Fig. 2a, b), and endogenous p53 was able to be co-immunoprecipitated with Lon under oxidative stress (Fig. 2c), suggesting that mitochondrial Lon interacts with p53 in vivo. To further confirm the finding, we performed Co-IP experiment using the isolated mitochondrial fraction. Consistently, the expression of p53 is increased in mitochondrial fraction when Lon is overexpressed; overexpression of p53 triggers its accumulation in mitochondrial fraction (Fig. 2d, left panel). Lon was able to be co-immunoprecipitated with p53 in mitochondrial fraction and vice versa (Fig. 2d, right panel). Furthermore, the His-tag and GST-tag pull-down assays confirmed a direct interaction between Lon and p53 in vitro (Fig. 2e). These data demonstrate that mitochondrial chaperone Lon interacts with p53 under oxidative stress.

### ATPase activity of Lon is required for the interaction with p53 by which attenuates apoptosis in mitochondria

**Fig. 3:**
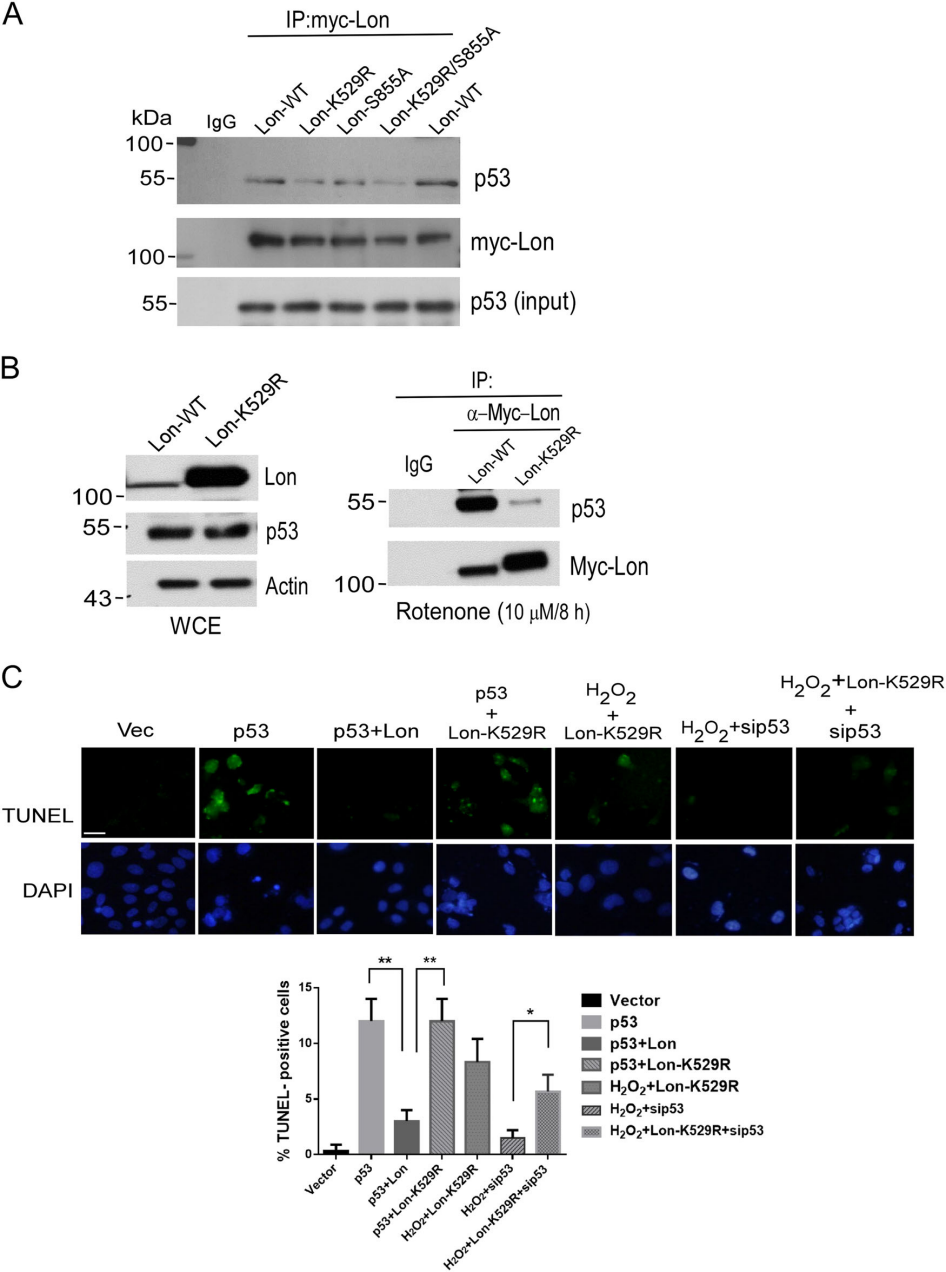
The chaperone activity of Lon is required for the interaction with p53 and the inhibition of p53-dependent apoptosis in cancer cells. **a** The K529 residue of mitochondrial Lon is required for the interaction with p53. Whole-cell lysates from HSC3 cells transfected with the plasmids encoding p53 and wild-type pcDNA3-Myc-Lon (Lon-WT), an ATPase mutant (LonK529R), a proteolytic mutant (LonS855A), or an ATPase/proteolytic mutant (LonK529R/LonS855A) were immunoprecipitated with anti-Myc antibodies. The immunoprecipitation complex was analyzed by immunoblotting using the indicated antibodies. **b** The K529 residue of mitochondrial Lon is required for the interaction with endogenous p53 under oxidative stress. Whole-cell lysates from HSC3 cells transfected with the plasmids encoding Lon-WT and LonK529R mutant were analyzed by western blotting using the indicated antibodies (left panel). Whole-cell lysates from HSC3 cells transfected with the plasmids encoding Lon-WT and LonK529R mutant under rotenone treatment (10 μM for 8 h) were immunoprecipitated with anti-Myc-Lon antibodies. The immunoprecipitation complex was analyzed by western blotting using the indicated antibodies. **c** K529 residue of mitochondrial Lon is required for the inhibition toward p53-dependent apoptosis under oxidative stress shown by TUNEL assay. Apoptosis in HSC3 cells was induced by p53 overexpression. HSC3 cells were transfected with sip53 and/or the plasmids encoding p53, wild-type pcDNA3-Myc-Lon, or an ATPase mutant of Lon (LonK529R). TUNEL assay was applied to examine the effect of the K529 residue of mitochondrial Lon on p53-induced apoptosis under oxidative stress (200 μM H_2_O_2_ for 4 h). TUNEL-positive cells (green fluorescence) were counted in the transfected cells. DAPI was used for nuclear staining. Scale bar, 20 μm. The error bars shown in the right panel represent the standard deviation from three different experiments. **p < 0.01

To explore that the chaperone activity of Lon is required for the interaction with p53, we used the ATPase mutant of Lon, Lon-K529R^23^, to examine whether the ATPase activity is critical to the interaction with p53. The association between Lon and p53 was examined by Co-IP experiment. The result showed that the Lon-K529R mutant significantly abolished the interaction with p53 but not in the protease mutant, Lon-S855A (Fig. 3a). Consistently, endogenous p53 was able to be co-immunoprecipitated with Lon-WT under rotenone treatment but not the Lon-K529R mutant (Fig. 3b), suggesting that the ATPase activity of mitochondrial Lon is required for the binding with p53. To confirm the chaperone activity of Lon is important in p53-mediated apoptosis regulation, TUNEL assay was performed by using the overexpression of Lon-K529R mutant. The TUNEL-positive cells in p53-overexpressed cells were significantly decreased when Lon was also overexpressed. However, the Lon-K529R mutant failed to inhibit p53-induced apoptosis. Similarly, the Lon-K529R mutant failed to inhibit H_2_O_2_-induced apoptosis (Fig. 3c). However, the TUNEL-positive signals were reduced when p53 was knocked down under H_2_O_2_ treatment and the positive signals induced by the overexpression of Lon-K529R mutant were rescued when p53 was knocked down under H_2_O_2_ treatment (Fig. 3c). These data showed that mitochondrial Lon interacts with p53 through its chaperone activity that is required to restrain p53-dependent apoptosis in cancer cells under oxidative stress.

### Increased chaperone Lon hampers the transcription-dependent apoptotic function of p53 under oxidative stress by retaining p53 in the mitochondria

**Fig. 4:**
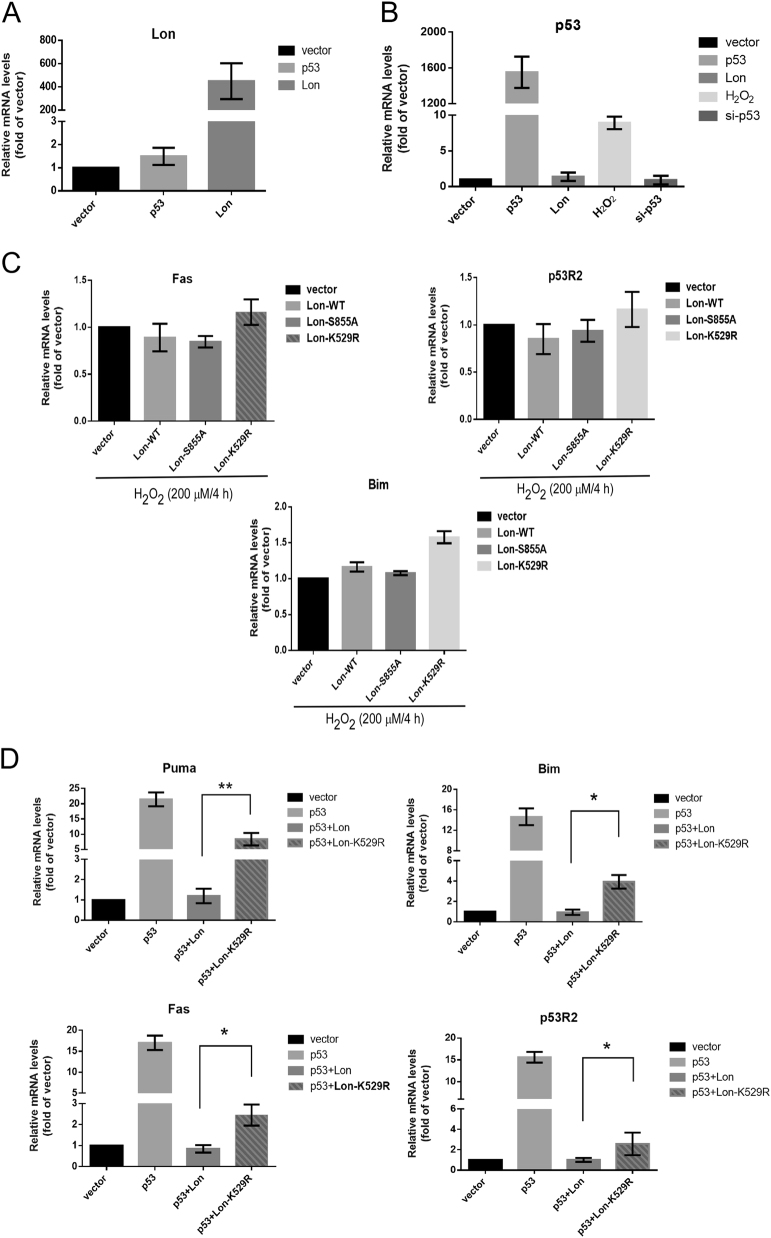
Increased mitochondrial Lon restrains the transcription-dependent function of p53 under oxidative stress through its chaperone activity. **a**, **b** The mRNA expression of Lon and p53 was analyzed by quantitative real-time PCR. **c** HSC3 cells were transfected with the plasmids encoding wild-type pcDNA3-Myc-Lon, a proteolytic mutant (LonS855A), or an ATPase mutant of Lon (LonK529R) under oxidative stress (200 μM H_2_O_2_ for 4 h). The mRNA expression of p53-targeted genes, Bim, Fas, and p53R2, was analyzed by quantitative real-time PCR. The results were presented as fold increase relative to vector-transfected cells (deliberately set to 1). Data are presented as mean ± SD of at least three independent experiments. The error bars shown in the panel represent the standard deviation from three different experiments. **d** HSC3 cells were transfected with the plasmids encoding p53 and/or wild-type pcDNA3-Myc-Lon or an ATPase mutant of Lon (LonK529R). The mRNA expression of p53-targeted genes, Puma, Bim, Fas, and p53R2, was analyzed by quantitative real-time PCR. The results were presented as fold increase relative to vector-transfected cells (deliberately set to 1). Data are presented as mean ± SD of at least three independent experiments. The error bars shown in the panel represent the standard deviation from three different experiments. *p < 0.05 and **p < 0.01.

Since p53 is known for its ability to orchestrate cell cycle and apoptosis by a transcription-dependent mechanism^24,25^, we examined whether the suppression of p53-dependent apoptosis by mitochondrial Lon is through affecting the distribution between nuclear and mitochondrial p53. Thus we checked whether the transcription-dependent function of nuclear p53 is affected by increased chaperone Lon. The mRNA expression of Lon gene was significantly increased when Lon was overexpressed (Fig. 4a). The mRNA expression of p53 gene was significantly increased when the cells were overexpressed by p53 and treated with H_2_O_2_, but not in Lon-overexpressed and p53-knocked-down cells (Fig. 4b). This result indicates that Lon overexpression is not able to affect p53 gene expression. Then we checked the expression of several p53-targeted genes, such as Puma/Bim in the intrinsic apoptosis pathway^24^, Fas that is involved in the extrinsic pathway^25^, and p53R2 that is required for mitochondrial DNA stability and cell protection from oxidative stress^26,27^.

The result showed that increased Lon-WT and the Lon-S855A mutant reduce the induction of p53-dependent apoptotic genes under oxidative stress. However, the overexpression of Lon-K529R mutant increased the expression of p53-dependent apoptotic genes under the same condition (Fig. 4c). Consistently, increased mitochondrial Lon reduced the induction of p53-dependent apoptotic genes when p53 was overexpressed. However, the Lon-K529R mutant failed to largely inhibit the expression of p53-targeted genes (Fig. 4d and Supplemental Figure S2). These results indicate that the ATPase activity of chaperone Lon indeed is important for p53-dependent apoptosis; the mechanism of apoptotic inhibition by increased Lon under oxidative stress is associated with sequestering some p53 in mitochondria that also lowers the nuclear distribution of p53.

### Chaperone Lon-mtHsp70 is required for mitochondrial p53 accumulation that is important to prevent the cytosolic distribution of p53 from proteasome-dependent degradation

**Fig. 5:**
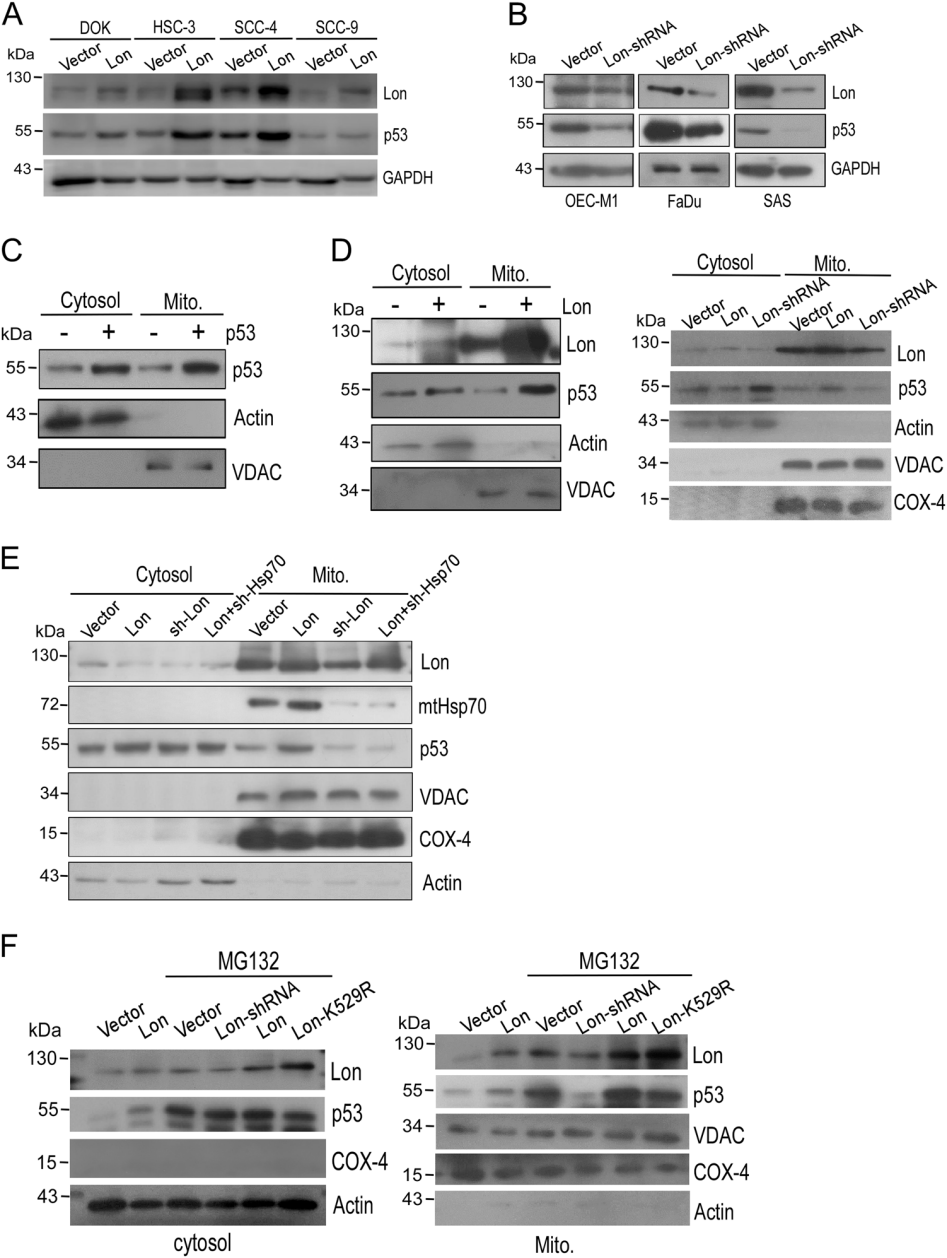
Chaperone Lon–mtHsp70 is required for mitochondrial p53 accumulation that is important to prevent the cytosolic distribution of p53 from proteasome-dependent degradation in cancer cells. **a**, **b** Mitochondrial Lon is important for the stability/level of p53 protein in cancer cells. For overexpression experiment, oral cancer cells were transfected with the plasmid encoding Myc-tagged Lon. For knocking down experiment, Lon expression was inhibited by Lon-shRNA transfection. Immunoblotting were performed using the indicated antibodies. **c** Immunoblotting analysis of increased p53 in mitochondrial fraction. p53 was overexpressed in OEC-M1 cells by transfected with the plasmid encoding p53. Immunoblotting were performed using the indicated antibodies. The purity of each cell fractions was monitored by immunoblotting for cytoplasmic (Actin) and mitochondrial (VDAC) markers. **d** Chaperone Lon is required for mitochondrial p53 accumulation. OEC-M1 cells were transfected with the plasmid encoding Myc-tagged Lon or Lon-shRNA. Immunoblotting were performed using the indicated antibodies. The purity of each cell fractions was monitored by immunoblotting for cytoplasmic (Actin) and mitochondrial (VDAC and COX-4) markers. **e** Chaperone Lon-induced mitochondrial p53 accumulation is dependent on mtHsp70. OEC-M1 cells were transfected with the plasmid encoding Myc-tagged Lon, Lon-shRNA, and/or mtHsp70-shRNA. Immunoblotting were performed using the indicated antibodies. The purity of each cell fractions was monitored by immunoblotting for cytoplasmic (Actin) and mitochondrial (VDAC and COX-4) markers. **f** The chaperone activity of Lon is required for mitochondrial p53 accumulation and the prevention of the cytosolic distribution of p53 from proteasome-dependent degradation. OEC-M1 cells were transfected with the plasmid encoding WT-Lon, Lon-shRNA, or the ATPase mutant of Lon (LonK529R). The transfected cells were treated with 10 μM MG132 for 2 h. Immunoblotting were performed using the indicated antibodies. The purity of each cell fractions was monitored by immunoblotting for cytoplasmic (Actin, left) and mitochondrial (VDAC and COX-4, right) markers

We found that the levels of p53 in cytosol and mitochondria were further increased when Lon was overexpressed in cells under oxidative stress (Fig. 1b), and the level of p53 in mitochondria was decreased when Lon was downregulated but not the level in cytosol under oxidative stress (Fig. 1c), suggesting that the mitochondrial accumulation and level of p53 is regulated by increased Lon under oxidative stress. Since previous findings showed that overexpression and knocked down of Lon both induce the production of ROS^12,28^, we tried to understand the mechanism of p53 accumulation induced by increased Lon without H_2_O_2_ treatment. Consistently, we observed that the protein level of p53 was increased when Lon was overexpressed in cancer cells (Fig. 5a), and knocked down of Lon caused a decrease in p53 level (Fig. 5b). We confirmed that p53 was accumulated in mitochondrial fraction when p53 was overexpressed in cells (Fig. 5c). p53 was increased in cytosol and mitochondrial fraction when Lon was overexpressed (Fig. 5d, left panel), and the level in mitochondria was decreased when Lon expression was inhibited by short hairpin RNA (shRNA; Fig. 5d, right panel). Intriguingly, we found that p53 still remained in the cytosol fraction when Lon was knocked down (Fig. 5d, right panel). These data suggest that Lon may be involved in the stability of p53 in the mitochondrial matrix, which may affect the distribution or transportation of p53 between cytosol and mitochondria. To test this idea, we knocked down mtHsp70 (mortalin) to examine the mechanism of p53 accumulation induced by increased Lon because mtHsp70 is involved in both protein import and folding process in mitochondrial protein homeostasis and is recently identified as a chaperone client of Lon^17^. We confirmed that p53 was increased in cytosol and mitochondrial fraction when Lon was overexpressed and the levels of p53 and mtHsp70 in mitochondria were decreased when Lon was knocked down by shRNA (Fig. 5e). However, the mitochondrial p53 accumulation induced by Lon overexpression was diminished when mtHsp70 was knocked down, suggesting that mtHsp70 is required for p53 accumulation in the mitochondria when Lon was overexpressed (Fig. 5e). Since we found that Lon-mtHsp70 is required for p53 accumulation in the mitochondria but not for the p53 level in the cytosol, we next asked whether the level of mitochondrial Lon regulates the distribution between cytosolic and mitochondrial p53. To answer this question, we first treated the Lon-knocked-down cells with a proteasome inhibitor, MG132, the level of p53 was gradually recovered, and increased in the cells (Supplemental Figure S3), indicating that p53 level regulated by Lon is mediated by the prevention of proteasome-dependent degradation in cytoplasm. Under MG132 treatment, p53 was increased in both cytosolic and mitochondrial fraction when Lon was overexpressed (revised Fig. 5f), suggesting that cytosolic and mitochondrial p53 are both affected by cytosolic proteasome-dependent degradation. Consistently, p53 was accumulated in the cytosol fraction under MG132 treatment when Lon, Lon-shRNA, or Lon-K529R mutant was overexpressed (Fig. 5f, left panel). However, p53 was decreased in the mitochondrial fraction when Lon was knocked down by shRNA or overexpression of the Lon-K529R mutant even under MG132 treatment (Fig. 5f, right panel). These results indicate that the chaperone activity of mitochondrial Lon and mtHsp70 are important to accumulate p53 in the mitochondria and to prevent the cytosolic distribution of p53 from proteasome-dependent degradation.

**Fig. 6:**
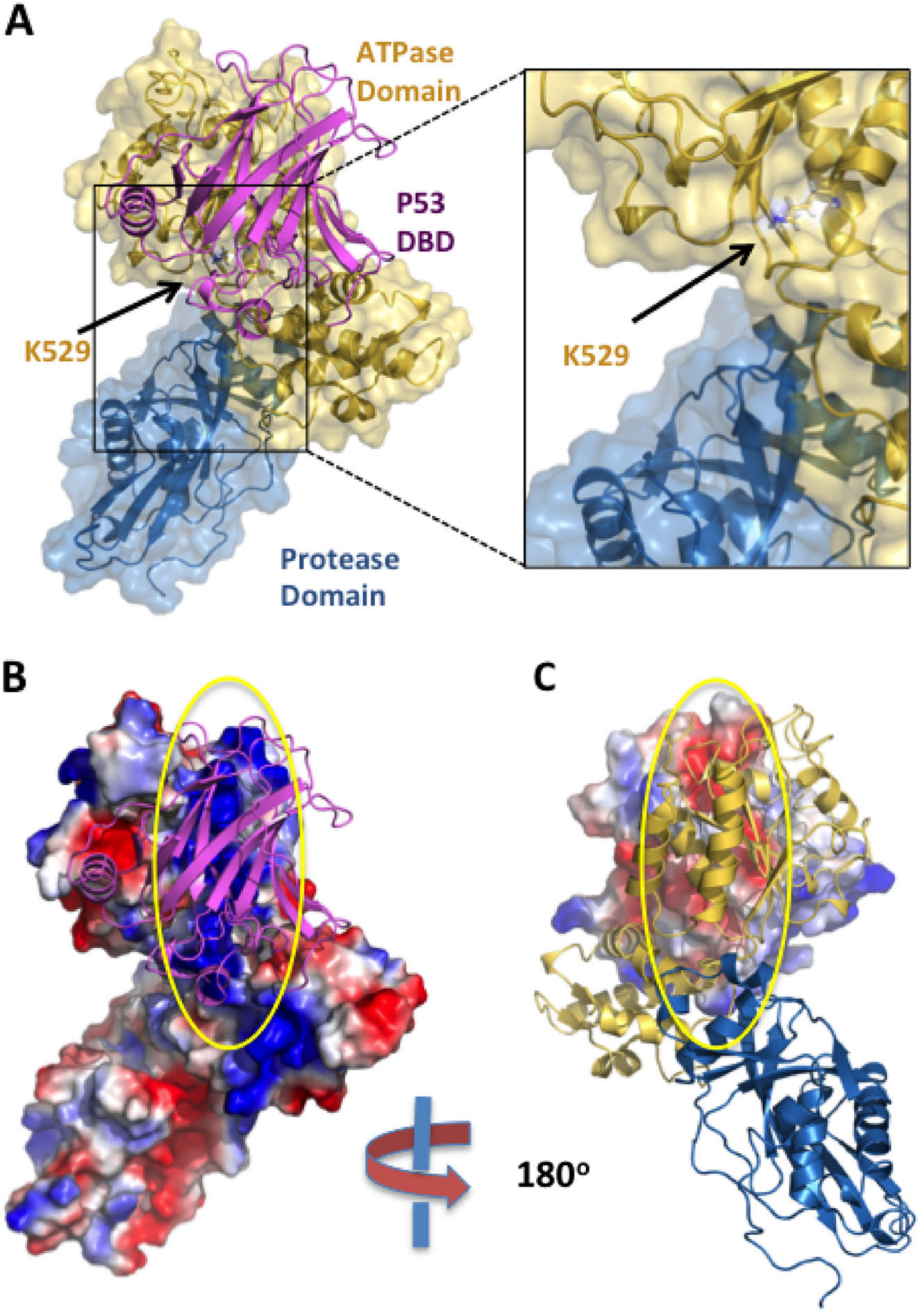
Model docking structure of mitochondrial Lon–p53 complex. **a** Ribbon representation of the structure of mitochondrial Lon–p53 complex. The protein structure is labeled along the sequence. Each domain is shown in a different color: ATPase domain in yellow, protease domain in blue, and p53 core domain in purple. K529 in ATPase pocket domain is indicated by arrow. **b** Surface-charge representation of mitochondrial Lon–p53 complex structure is shown. Basic, acidic and uncharged surface regions are colored blue, red and white, respectively. p53 core domain is represented by Ribbon in purple. **c**. Structure representation of the mitochondrial Lon–p53 complex. The same cartoon as **b** after an 180^o^ rotation around the vertical axis. Mitochondrial Lon is represented by Ribbon and p53 core domain is represented by surface-charge model. Basic, acidic, and uncharged surface regions are colored blue, red, and white, respectively

To understand the interaction between mitochondrial Lon and p53, we tried to build a complex model structure by using protein–protein docking and the information-driven approach. Indeed, the most stable complex model showed that p53 core structure binds into a prominent valley formed between the ATPase domain and alpha-subdomain of mitochondrial Lon (Fig. 6a, b). This observation is consistent with a previous study which showed that the ATP-dependent helicase domain of SV40 Large T-antigen bind to the core structure (DNA-binding domain) of p53^29^. Since the K529R mutant of Lon abolished the ability to hydrolyze ATP (ATPase activity) but retained the ability to bind ATP^23^, the conformational change of the mutant will be inhibited when ATP accessed into the ATP pocket and occupied the pocket, which may explain the steric effect of the ATPase mutant of chaperone Lon on the binding with p53. In addition, surface-charge representations of the Lon–p53 complex model structure were built (Fig. 6b, c). The structure shows that the surface-charge property in the p53-binding valley of Lon is positively charged (Fig. 6b), and the charge property of p53 in the Lon-binding patches is negatively charged (Fig. 6c). The result implies that electrostatic forces may mediate the interactions between mitochondrial Lon and p53 core structure.

### The level of cytoplasmic p53 protein correlates the level of mitochondrial Lon in oral cancer patients

**Fig. 7:**
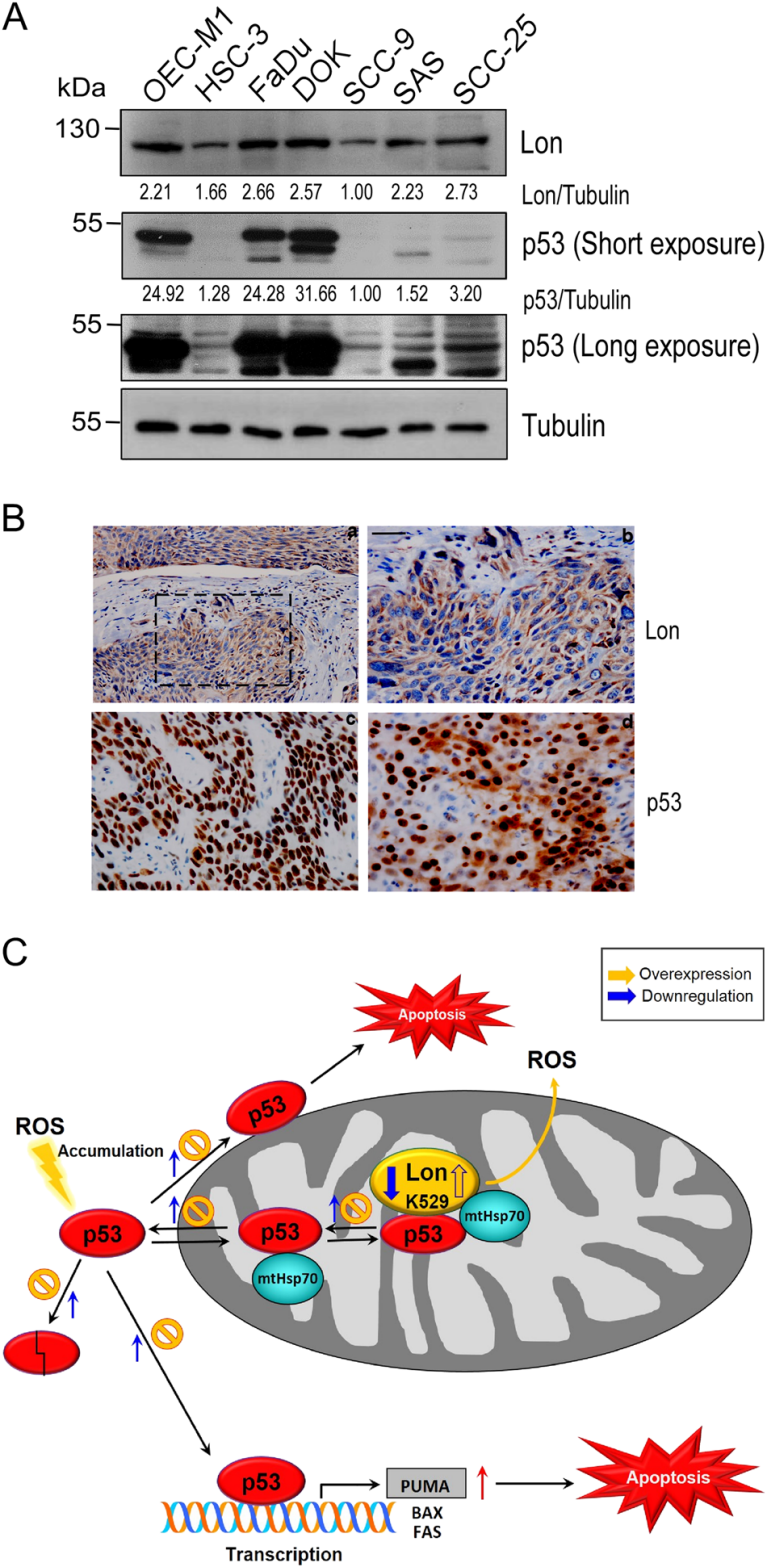
The level of cytoplasmic p53 protein correlates the level of mitochondrial Lon in oral cancer. **a** The protein level of Lon and p53 in oral cancer cell lines. The extracts of oral cancer cell lines were immunoblotted with the indicated antibodies and antibody to Tubulin as a loading control. **b** Immunohistochemical analysis of Lon and p53 expression in OSCC patients. Representative immunohistochemical analysis of Lon and p53 was performed by using paraffin-embedded sections of OSCC. The representative results shown here are positive staining of Lon (a, 200×; b, 400×), nuclear staining of p53 (**c**), and nuclear/cytoplasmic staining of p53 (**d**) in oral cancer tissues. The microscopic magnification of p53 staining was 400×. Scale bar, 50 μm. **c** Model of p53 accumulation in mitochondria and apoptosis inhibition by chaperone Lon in cancer cells. Upon Lon overexpression and/or oxidative stress, mitochondrial Lon binds with p53 and induces the accumulation of p53 in the matrix through its chaperone activity (residue K529) and inhibits p53-mediated apoptosis including transcription-independent and -dependent mechanisms. Mitochondrial Lon sequesters p53 to inhibit the opening of MPTP on the outer membrane and the MPTP–cyclophilin D complex on the inner membrane. Meanwhile, mitochondrial Lon retains p53 in the matrix to reduce the transcription-dependent function of nuclear distribution of p53. The stability/level of cytosolic p53 is increased by the prevention of proteasome-dependent degradation through sequestering p53 by Lon-mtHsp70 in mitochondrial matrix

We found that total p53 level is controlled by the level of mitochondrial Lon in several cancer cell lines (Fig. 5a, b) and the mitochondrial accumulation of p53 is regulated by Lon (Fig. 5e, f), suggesting that the cytoplasmic level (includes mitochondrial fraction) of p53 protein correlates the level of mitochondrial Lon. We then examined whether p53 stability/level regulated by mitochondrial Lon is clinically relevant. First, we found that the expression of Lon protein correlates with that of p53 in seven oral cancer cell lines (Fig. 7a). To link the clinical significance of increased Lon and p53 level, 123 samples of tumor tissues from oral squamous cell carcinoma (OSCC) patients were used to determine Lon and p53 expression pattern by immunohistochemical (IHC) analysis. The main clinicopathological characteristics of the 123 patients of this study are detailed in Table S1. Mitochondrial Lon was found in the cytoplasm of cancer cells (a and b in Fig. 7b); p53 was identified in nucleus only (c in Fig. 7b) or in both nucleus and cytoplasm of cancer cells (d in Fig. 7b), which are consistent with our previous reports^12,30^. The high expression (IHC level, median and strong) of Lon was observed in the majority of OSCC tumor tissues (85/123, 69.1%); the high expression of p53 showed a nearly half ratio in tumor tissues (64/123, 52.0%) (Table S2). We next examined the association between Lon and p53 expression in OSCC tissues using Fisher’s exact test and measured the correlation in contingency table. The results showed that p53 expression in either nucleus/cytoplasm or nucleus only shows no significant correlation with Lon expression (*P* = 0.879 and *P* = 0.264, respectively, Table S2 and S3). However, p53 simultaneous expression in cytoplasm showed a significant correlation with Lon expression (*p* = 0.047, Table 1). Consistently, when the expression pattern was categorized into three groups: strong, median, and weak expression, the correlation between Lon and p53 expression in nucleus/cytoplasm is statistically significant (*p* = 0.05) with a Cramer’s V coefficient of 0.44 (Table S4) but not in nucleus or cytoplasm or in nucleus only (Table S5 and S6). Taken together, these result indicated that cytoplasmic p53 protein is correlated with the level of Lon protein in OSCC patients.

**Table 1:** The contingency table shows a positive association between Lon and p53 protein, based on 28 patients with p53 protein's simultaneous expression in cell cytoplasm

		Lon				Fisher, *p*
		None	Weak	Median	Strong	
p53	None	0	0	0	0	0.047
	Weak	0	2	0	1	
	Median	0	0	0	5	
	Strong	2	1	8	9	

## Discussion

In this study, we have shown that mitochondria Lon interacts with p53 and retains it in mitochondrial matrix to restrain the apoptosis induced by oxidative stress. The ATPase mutant of Lon decreases the interaction with p53 and fails to inhibit apoptosis under oxidative stress. This study reveals that Lon overexpression inhibits apoptosis through the chaperone activity by interacting with and sequestering p53 in mitochondria.

Molecular chaperones of HSP family play important roles in promoting cell survival and tumor growth^18,31^. We previously identified a number of candidate client proteins of mitochondrial chaperone Lon by using a proteomic approach. We identified NDUFS8, Hsp60, and mtHsp70 are client proteins of mitochondrial Lon^12,17^. Mitochondrial Lon physically interacts with Hsp60–mtHsp70 complex and the protein stability/level of Hsp60 and mtHsp70 depends on the level of Lon under oxidative stress^17^. Hsp60 binds p53 to restrain its apoptosis function: the depletion of Hsp60 increases the level of p53 but does not affect MDM2 level^16^, suggesting that there is an unknown mechanism to regulate the p53 protein level beyond nucleus and cytoplasm. Mitochondrial Hsp60 inhibits apoptosis by antagonizing cyclophilin D-dependent mitochondrial permeability transition^32^, increasing the stabilization of survivin and restraining p53 function^16^. The scenarios for the mechanism underlying Lon-mtHsp70-inhibited apoptosis through binding with and stabilizing p53 are described (Fig. 7c). We found that increased mitochondrial Lon inhibits p53-mediated apoptosis including transcription-independent and -dependent mechanism. p53-mediated cell death is involved in transcription-dependent and -independent regulation^24,33,34^. Regarding transcription-independent mechanism, in response to oxidative stress, the cytoplasmic pool of p53 protein localizes to the outer membrane of mitochondria where it activate transcription-independent apoptosis by physically inhibiting antiapoptotic members (Bcl2, BclxL) as well as activating proapoptotic members (Bak, Bax) of mitochondrial outer membrane permeabilization (MOMP) regulators^5,34^. To our knowledge, the present study for the first time demonstrates that p53 is bound by Lon in the mitochondrial matrix to control MOMP and apoptosis. Indeed, we observed that increased mitochondrial Lon rescued Δψm and the release of cytochrome C induced by p53. This finding is consistent with the observation that p53 accumulates in the mitochondrial matrix and triggers MPTP opening and necrosis by interaction with cyclophilin D under oxidative stress^2^. MPTP is a regulated protein channel spanning the inner and outer mitochondrial membranes. Previous reports showed that matrix Lon interacts with proteins located at inner membrane, such as prohibitin, COX, and NDUFS8^12,17,35,36^. We suggest that Lon may serve as a recruiter complex center to trap p53 to disrupt the interaction with cyclophilin D, by which it could prevent apoptosis by inhibiting the opening of MPTP, high levels of cytosolic Ca^2+^, and ROS accumulation during oxidative stress.

This study indicates that the chaperone activity of mitochondrial Lon is important to control p53 protein level by translocating and sequestering p53 in mitochondria under oxidative stress. Consistently, the mutations in the AAA+ domain of Lon caused the aggregation of mtDNA-encoded cytochrome C oxidase subunit II, which reduces the function of mitochondrial respiration in CODAS syndrome patients^37^. Thus the possible mechanism underlying Lon-mediated stabilization of p53 will be controlling p53 translocation into mitochondria. Accumulating studies have shown that at least three different mechanisms were described by which p53 translocates into the matrix of mitochondria, including the two classical import systems utilizing mitochondrial targeting sequences (MTS) or chaperone carriers and one mechanism that involves redox/respiration-dependent import system^38,39^. However, no MTS were found in the N- or C-terminal domain of p53 protein. Thus it is worth attracting more attention that p53 translocates into the matrix by the mechanism of chaperone carriers. Indeed, the mitochondrial trafficking of cytosolic p53 is mediated by mtHsp70/Tid-1 complex under DNA damage and hypoxia^40,41^. Our previous work showed that mtHsp70/Hsp60 complex act as Lon-associated proteins and their protein level and stability are dependent on Lon^17^. Therefore, increased mitochondrial Lon may promote the translocation of p53 into mitochondria by stabilizing the chaperone carriers, Hsp60/mtHsp70/Tid-1 complex. Consequently, mitochondrial Lon-mtHsp70 restrains p53-dependent apoptosis under stress, including the function of nuclear p53-dependent transcription and cytosolic p53-dependent mitochondria targeting.

In summary, we identified and validated p53 as a chaperone client of Lon along with Hsp60 and mtHsp70. This study for the first time reported that the function of p53 translocated into mitochondrial matrix in apoptosis regulation. Mitochondrial Lon retains p53 in the mitochondria matrix through its chaperone activity and inhibits p53-mediated apoptosis by transcription-independent and -dependent mechanisms. We have shown that mitochondria Lon interacts with p53 and retains it in mitochondrial matrix to restrain the apoptosis induced by oxidative stress. The ATPase mutant of Lon decreases the interaction with p53 and fails to inhibit apoptosis under oxidative stress. Our studies will provide new insights into the chaperone function of Lon in apoptotic cell death exerted by directly sequestering p53 in mitochondria and will allow us to understand that targeting the chaperone activity of mitochondrial Lon increases the efficacy of p53-induced apoptosis in cancer therapy.

## Materials and methods

### Patients and clinical sample

Tissue specimens of 123 patients with OSCC were chosen for IHC analysis based on availability of archival human oral tissue blocks from diagnostic resection specimens in the Departments of Pathology at Mackay Memorial Hospital, Taipei, Taiwan with approval from the Institutional Review Board (IRB number: 13MMHIS188). The main clinical characteristics of the 123 patients selected for this study are detailed in Table S1. All experiments were performed in accordance with relevant guidelines and regulations. The levels of Lon and p53 protein expression are categorized into no available, low, median, and strong based on the scores of IHC staining.

### Cell culture and cell treatment

293, 293T, HSC3, FADU, and DOK (dysplastic oral keratinocyte) cells were cultured in medium containing Dulbecco’s modified Eagle’s essential medium (DMEM) (GIBCO, New York, NY, USA), supplemented with 5% fetal bovine serum (FBS) and 5% super calf serum and penicillin–streptomycin (50 U/ml, Sigma, St. Louis, MO, USA) in a 5% CO_2_/95% air atmosphere. SCC-9, SCC-25, and SAS cells were cultured in medium containing a 1:1 mixture of DMEM/F12 medium, supplemented with 10% FBS. OEC-M1 cells were cultured in medium containing RPMI 1640 medium, supplemented with 10% FBS. Stable cell lines expressing Lon-shRNA were generated by retroviral infection using pMKO vector. Briefly, the plasmids, Lon-Sh1/2-pMKO-puro for stable knockdown cells, along with packaging plasmid gag-pol and envelope plasmid VSV-G, were transfected into 293T cells by Lipofectamine™ 2000 (Invitrogen, Carlsbad, CA, USA). The cells were incubated for 24 h and the medium was changed to remove remaining transfection reagent. Retroviral supernatant was collected at 24 and 48 h post-transfection and used to infect the target cells 293T for 48 h. Polybrene (hexadimethrine bromide) was added to the medium for improving infection efficacy. Puromycin (Sigma-Aldrich) was used to select the successfully infected cells at a final concentration of 2 µg/ml and the survived cells were collected to check the expression of human Lon by western blotting. Lon-shRNA: 5**′**-GAAAGUUCGUCUCGCCCAGCC-3**′** (sh-1) or 5**′**-AGGAGCAGCUAAAGAUCAUCA-3**′** (sh-2)^12,17^. Cultured cells were treated with hydrogen peroxide (200 µM H_2_O_2_, Sigma-Aldrich) for 4 h at 37 °C.

### Antibodies

Antibodies to human Lon was produced as described previously^28^. Antibodies used in this study were purchased as indicated: antibody to p53 and Flag from Sigma; Myc (9E10) from Millipore; α-tubulin (ab4074) and COX4 (ab16056) from Abcam (Cambridge, MA, USA); IkB alpha and VDAC from Cell Signaling Technology (Beverly, MA, USA); Bcl-2, Bax, and cytochrome C from Santa Cruz Biotechnology, Inc. (Santa Cruz, CA, USA); GAPDH and beta-actin from GenTex (Hsinchu, Taiwan).

### Western blot analysis

Western blot analysis was performed as described previously^12,17^.

### Isolation of mitochondria fraction

Mitochondrial fraction was isolated by the Mitochondria Isolation Kit for Cultured Cells (Thermo Fisher Scientific, 89874) according to the manufacturer’s instructions.After collecting the mitochondria fraction from cells, the mitochondrial pellets were resuspended with Lysis buffer, NETN buffer with protease inhibitors (100 mM PMSF, 5 µg/ml aprotinin, 5 µg/ml leupeptin) on ice for 30 min and centrifuged at 12,000 × *g* for 15 min. Then the supernatant was collected and applied for subsequent western blotting.

### Co-immunoprecipitation

Cells were lysed in NETN (150 mM NaCl, 1 mM EDTA, 20 mM Tris-Cl (pH 8.0), 0.5% NP-40) containing protease and phosphatase inhibitors (1.0 mM sodium orthovanadate, 50 μM sodium fluoride). Immunoprecipitation was performed by incubating primary antibodies with cell lysates at 4 °C for overnight, followed by the addition of secondary antibody and protein A/G-agarose (Calbiochem) for two additional hour with slow agitation and centrifugation for 15 s. The pellets were washed three times with NETN containing protease inhibitor cocktail (Roche) buffer and examined for binding partners by western blotting.

### Immunofluorescence

Cells were plated on glass coverslips placed in a 12-well culture dish. When cells had attached to the surface and spread well, they were washed with cold phosphate buffered saline (abbreviated PBS) and then fixed with pre-cold methanol/ acetone (1:1, v/v) mixture for 15 minutes at room temperature. Fixed cells were washed with PBS and permeabilized with 0.5 % (v/v) Triton X-100 in PBS for 15 min at room temperature. Cells on coverslips were incubated with indicated antibodies: anti-Lon (1:400) and anti-p53 (1:200) overnight at 4 °C. The following day, fixed cells were washed three times with 0.5% Triton X-100 in PBS and incubated with Alexa 488-conjugated and Alexa-594 conjugated anti-mouse or anti-rabbit secondary antibodies. Finally, coverslips were mounted by ProLong® Gold Antifade Reagent with DAPI (Invitrogen, Carlsbad, CA) for room temperature 10 min. Fluorescent images were acquired by by an Olympus BX51 fluorescence microscopy.

### Apoptosis assay

Apoptosis was analyzed by TUNEL staining or MMP measurement. Cell apoptosis was detected by TUNEL assay according to the manufacturer’s instructions (TaKaRa BIO, Shiga, Japan) and was performed as described previously^12,42^. Apoptotic cells also were analyzed by flow cytometry after DiOC6(3) (AnaSpec, Inc) staining. Flow cytometry and data analysis were carried out on a FACSCalibur instrument (Becton Dickinson), excitation = 488 nm; emission = 530 nm (F1), using the program Lysis.

### Reverse transcription-PCR (RT-PCR)

Total RNA was extracted using Trizol reagent (Invitrogen) and reverse-transcribed using Maxima First Strand cDNA Synthesis Kit for RT-qPCR (Thermo Fisher Scientific, USA). The resulting cDNA was used as the template for PCR reactions. Real-time PCR reactions were performed using the ABI Step One Plus Real-Time PCR System (Applied Biosystems, Foster City, CA, USA) using SYBR Green PCR Master Mix (Thermo Fisher Scientific, USA) and relative quantification was performed using the comparative 2^−^^ΔΔCT^ method. The sets of forward and reverse primers, the corresponding PCR conditions, and the lengths of PCR products were described as follows: Lon (5**′**-GTCATGGATGTTGTGGACGA-3**′,** 5′**-**GTAGTTGCGGGTGACATTGA**-**3′**)**; p53 (5**′**-GGCTCTGACTGTACCACCATCCA-3**′**, 5**′**-GGCACAAACACGCACCTCAAAG-3**′**); Fas (5**′**-TCTGGTTCTTACGTCTGTTGC-3**′**, 5**′**-CTGTGCAGTCCCTAGCTTTCC-3**′**); Bax (5**′**-TGCTTCAGGGTTTCATCCAG-3**′**, 5**′**-GGCGGCAATCATCCTCTG-3**′**); Puma (5**′**-GAAGAGCAAATGAGCCAAACG-3**′**, 5**′**-GGAGCAACCGGCAAACG-3**′**); p53R2 (5**′**-GAGGCTCGCTGTTTCTATGG-3**′**, 5**′**-ATCTGCTATCCATCGCAAGG-3**′**); Bim (5**′**-TGGCAAAGCAACCTTCTGATG-3**′**, 5**′**-GCAGGCTGCAATTGTCTACCT-3**′**). All the PCR reactions were started at 94 °C for 5 min and terminated at 72 °C for 5 min. Finally, the data were analyzed using the StepOne Software v2.3. Differential RNA expressions between various samples were calculated using β-actin as an internal control.

### Protein structure and modeling

Structural modeling of human Lon was built by using I-TASSER package^43^ by iterative fragment assembly simulation for protein 3D structure prediction and refinement. The best predicted structure was used for further application. Docking of p53 DNA-binding domain (PDB code: 1TSR) to the structure of human Lon ATPase domain from predicted model was initially carried out using the ZDOCK server^44^, which employs rigid-body docking and utilizes a scoring function based on pairwise shape complementarity, desolvation, and electrostatic energies. No residue constraints were supplied as inputs for docking calculation. The structure of human Lon ATPase domain was assigned as the receptor in the docking calculation. During rigid-body energy minimization, >500 structures were calculated and the 100 best structures based on the intermolecular energy were used for semiflexible simulated annealing. Docked structures corresponding to the 100 best structures with the lowest intermolecular energies were generated. Finally, the top 1 docking models predicted by ZDOCK data was selected for our biochemical elucidation.

### Statistical methods

We examined the significance of the IHC staining association between Lon and p53 protein using Fisher’s exact test and considered a statistical significance if a *p* value was ≤0.05. We measured the correlation in contingency table using Cramer’s V coefficient, which was calculated by CramersV function in R-based lsr package. All data were analyzed using the R statistical software (version 3.1.1). Parametric Student’s t test was used to judge the significance of difference between conditions of interest. In general, a *p* value of <0.05 was considered as statistically significant (*p < 0.05, **p < 0.01, and ***p < 0.001).

## Electronic supplementary material

Supplementary Data
