# Supplementary material for: Mitochondrial Lon sequesters and stabilizes p53 in the matrix to restrain apoptosis under oxidative stress via its chaperone activity
Source: Cell Death Dis. 2018 Jun 13;9(6):697. doi: 10.1038/s41419-018-0730-7 (PMC5998145; doi:10.1038/s41419-018-0730-7)
Supplement: Supplementary file 1 — Supplementary Data [file 41419_2018_730_MOESM1_ESM.docx]

**Mitochondrial Lon sequesters p53 in the matrix to restrain apoptosis under oxidative stress via its chaperone activity**

Ya-Ju Sung^1,2^#, Ting-Yu Kao^2^#, Cheng-Liang Kuo^1^, Chi-Chen Fan^2,3^, An Ning Cheng^1^, Wei-Cheng Fang^1^, Han-Yu Chou^1^, Yu-Kang Lo^1^, Chung-Hsing Chen^1^, Shih Sheng Jiang^1^, I-Shou Chang^1^, Chun-Hua Hsu^4,5^, Jin-Ching Lee^6,7,8^, and Alan Yueh-Luen Lee^1,6^*

**
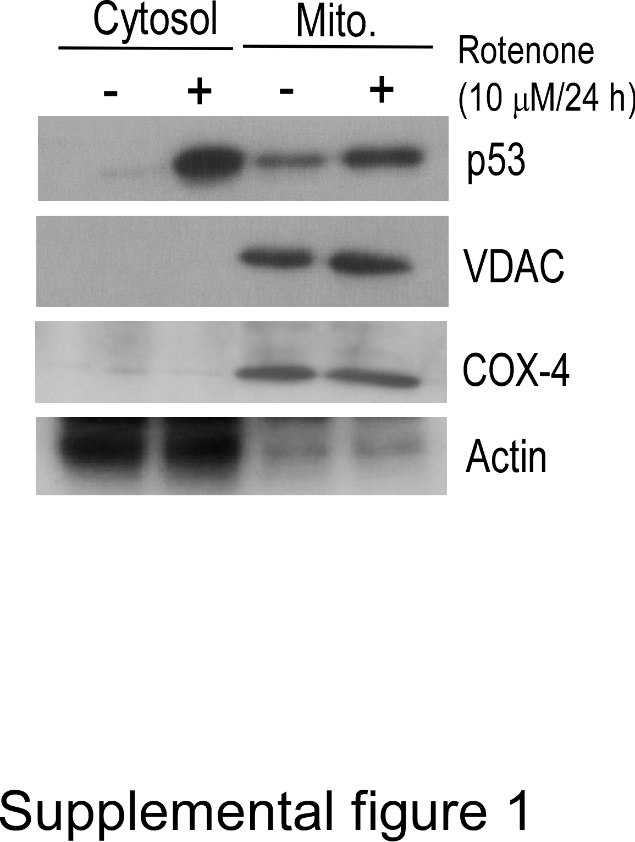
**

**Figure S1. Increased Lon increases the accumulation of mitochondrial p53 and inhibits p53-dependent apoptosis under oxidative stress in cancer cells**

HSC-3 cells were treated with 10 μM rotenone for 4 hr. Immunoblotting were performed using the indicated antibodies. The purity of each cell fractions was monitored by immunoblotting for cytoplasmic (Actin) and mitochondrial (VDAC and COX-4) markers.

**
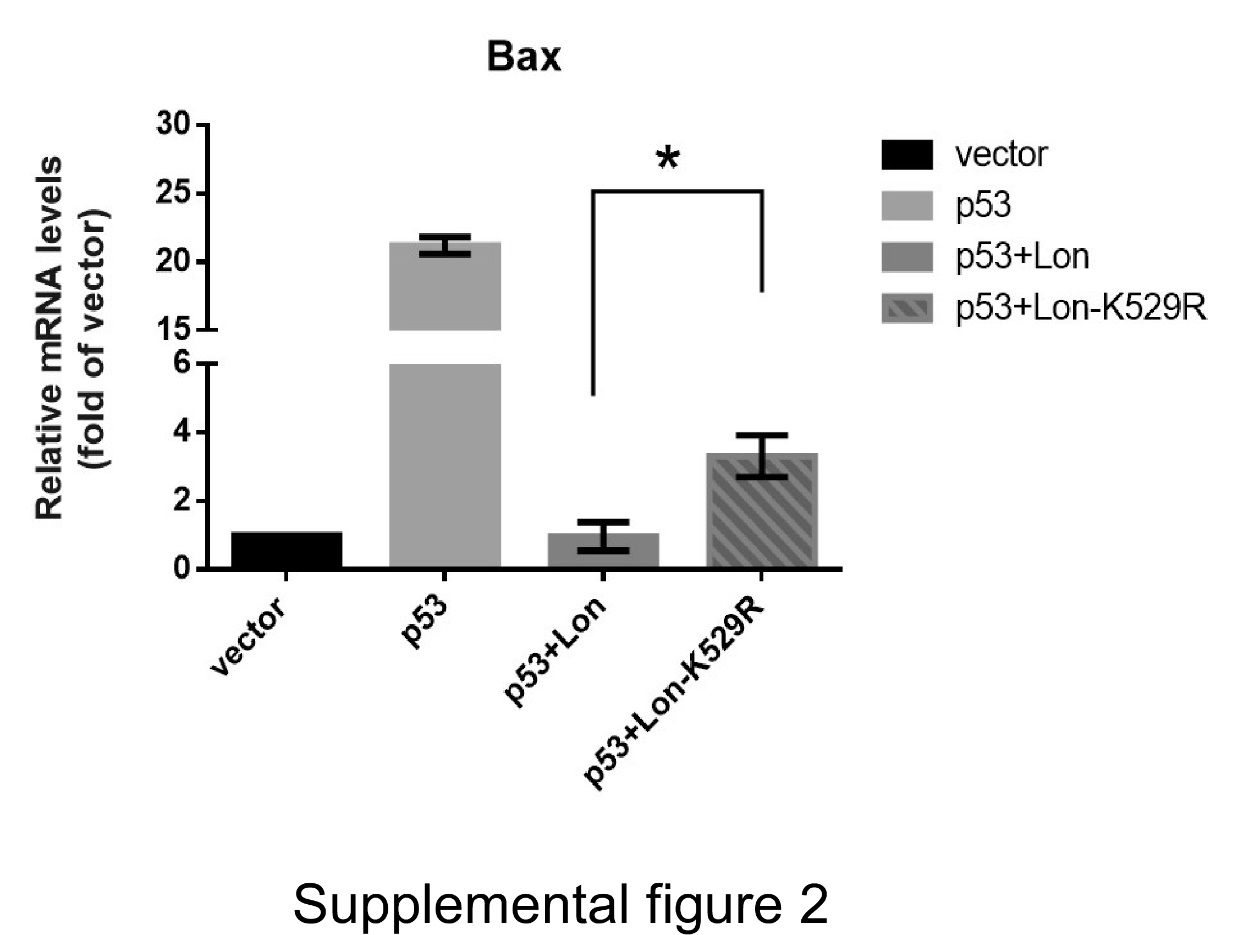
**

**Figure S2. Increased mitochondrial Lon restrains the transcription-dependent function of nuclear p53 through its chaperone activity**

The mRNA expression of p53-targeted gene, Bax, was analyzed by quantitative real-time PCR. The results were presented as fold increase relative to vector-transfected cells (deliberately set to 1). Data are presented as mean ± SD of at least three independent experiments. The error bars shown in the panel represent the standard deviation from three different experiments. *p < 0.05.

**
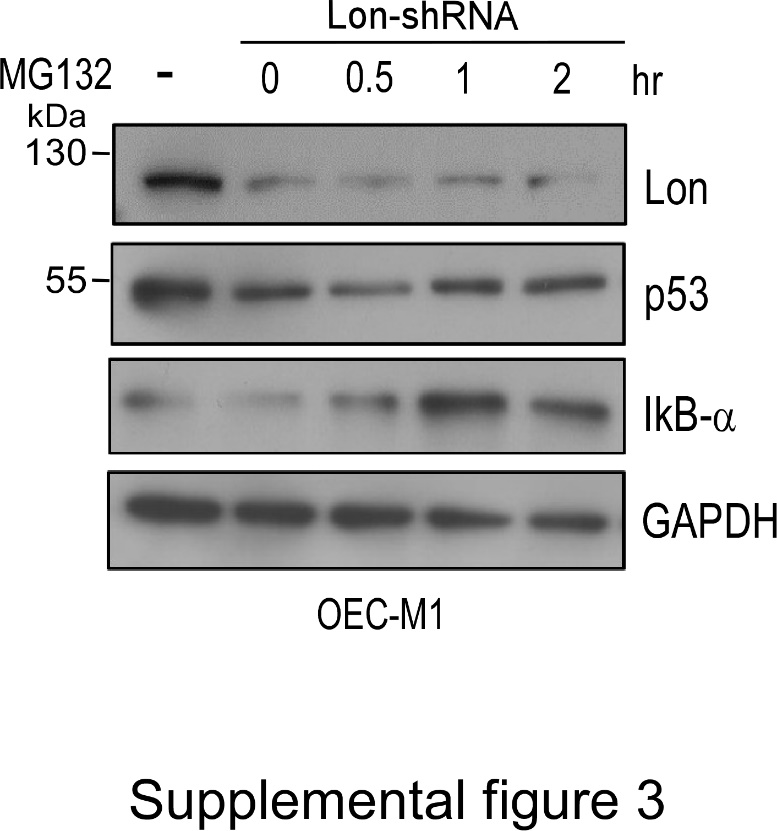
**

**Figure S3. Mitochondrial Lon contributes to the stability of p53. MG132 (proteasome inhibitor)-chase experiments of p53 in OEC-M1 shLon cells.**

OEC-M1 cells transfected with shLon (Lon-shRNA) were treated with 10 μM MG132 for 0.5, 1, 2 h. The level of p53 was determined by Western blotting. GAPDH was used as a loading control. The level of IkB-α was used as a control for MG132 treatment.

**Supplementary Data**

**Supplementary Table 1.** Clinicopathological characteristics in OSCC patients (*n=*123)

| Variables | | Number of patients (%) | |
| --- | --- | --- | --- |
| Age (years old) | |  |  |
|  | $\leq$ 62 | 69 | (56.1%) |
|  | $>$ 62 | 54 | (43.9%) |
| Gender | |  |  |
|  | Female | 7 | (5.7%) |
|  | Male | 116 | (94.3%) |
| Alcohol drinking | |  |  |
|  | No | 38 | (30.9%) |
|  | Yes | 85 | (69.1%) |
| Betel quid chewing | |  |  |
|  | No | 90 | (73.2%) |
|  | Yes | 33 | (26.8%) |
| Cigarette smoking | |  |  |
|  | No | 55 | (44.8%) |
|  | Yes | 68 | (55.2%) |
| Differentiation | |  |  |
|  | Well / Moderate | 110 | (89.4%) |
|  | Poor | 13 | (10.6%) |
| Tumor Size | |  |  |
|  | $\leq$ 2cm | 70 | (56.9%) |
|  | $>$ 2cm | 53 | (43.1%) |
| Lymph node metastasis | |  |  |
|  | No | 105 | (85.3%) |
|  | Yes | 18 | (14.7%) |
| TNM stage | |  |  |
|  | In situ | 3 | (2.4%) |
|  | I, | 13 | (10.6%) |
|  | II | 8 | (6.5%) |
|  | III | 11 | (9.0%) |
|  | IV | 86 | (69.9%) |
|  | No information | 2 | (1.6%) |
| Treatment |  |  |  |
|  | None | 6 | (4.9%) |
|  | RT | 25 | (20.3%) |
|  | CT | 48 | (39.0%) |
|  | CT+RT | 44 | (35.8%) |
| Recurrence |  |  |  |
|  | No | 81 | (65.9%) |
|  | Yes | 42 | (34.1%) |
| Death |  |  |  |
|  | No | 61 | (49.6%) |
|  | Yes | 62 | (50.4%) |

**Supplementary Table 2.** The contingency table shows no association between Lon and p53 protein, based on overall 123 patients.

|  |  | *Lon* | | | | Fisher, *P* |
| --- | --- | --- | --- | --- | --- | --- |
|  |  | None | Weak | Median | Strong |  |
| *p53* | None | 4 | 5 | 4 | 13 | 0.879 |
|  | Weak | 5 | 4 | 11 | 13 |  |
|  | Median | 2 | 2 | 6 | 9 |  |
|  | Strong | 7 | 9 | 13 | 16 |  |

**Supplementary Table 3.** The contingency table shows no association between Lon and p53 protein, based on 69 patients with p53 protein expression only in cell nucleus.

|  |  | *Lon* | | | | Fisher, *P* |
| --- | --- | --- | --- | --- | --- | --- |
|  |  | None | Weak | Median | Strong |  |
| *p53* | None | 0 | 0 | 0 | 0 | 0.264 |
|  | Weak | 5 | 2 | 11 | 12 |  |
|  | Median | 2 | 2 | 6 | 4 |  |
|  | Strong | 5 | 8 | 5 | 7 |  |

*NA: not available

**Supplementary Table 4.** The contingency table shows a positive association between Lon and p53 protein, based on 28 patients with p53 protein simultaneous expression in cell cytoplasm. Cramer’s V coefficient is presented in parentheses.

|  |  | *Lon* | | | Fisher, *P* |
| --- | --- | --- | --- | --- | --- |
|  |  | Weak | Median | Strong | (Cramer’s V) |
| *p53* | Weak* | 2 | 0 | 1 | 0.050 |
|  | Median | 0 | 0 | 5 | (0.437) |
|  | Strong | 3 | 8 | 9 |  |

*: The weak expression includes none and weak staining in IHC level.

**Supplementary Table 5.** The contingency table shows no association between Lon and p53 protein, based on overall 123 patients. Cramer’s V coefficient is presented in parentheses.

|  |  | *Lon* | | | Fisher, *P* |
| --- | --- | --- | --- | --- | --- |
|  |  | Weak | Median | Strong | (Cramer’s V) |
| *p53* | Weak* | 18 | 15 | 26 | 0.778 |
|  | Median | 4 | 6 | 9 | (0.085) |
|  | Strong | 16 | 13 | 16 |  |

*: The weak expression includes none and weak staining in IHC level.

**Supplementary Table 6.** The contingency table shows no association between Lon and p53 protein, based on 69 patients with p53 protein expression only in cell nucleus. Cramer’s V coefficient is presented in parentheses.

|  |  | *Lon* | | | Fisher, *P* |
| --- | --- | --- | --- | --- | --- |
|  |  | Weak | Median | Strong | (Cramer’s V) |
| *p53* | Weak* | 7 | 11 | 12 | 0.215 |
|  | Median | 4 | 6 | 4 | (0.208) |
|  | Strong | 13 | 5 | 7 |  |

*: The weak expression includes none and weak staining in IHC level.
